# Editorial: Exploring miRNA roles in neuroinflammation and brain aging: mechanisms and therapeutic potential

**DOI:** 10.3389/fimmu.2026.1959643

**Published:** 2026-08-13

**Authors:** Ken-Ichiro Fukuchi, Junling Yang, Joanna Williams

**Affiliations:** 1 Department of Cancer Biology and Pharmacology, University of Illinois College of Medicine Peoria, Peoria, IL, United States; 2 Department of Anatomy, University of Otago, Dunedin, New Zealand

**Keywords:** Alzheimer disease, biomarker, microglia, microRNA, multiple sclerosis, neuroinflamamation, pyroptosis, spinal cord injury

MicroRNAs (miRNAs) are small non-coding RNAs that regulate gene expression. Emerging evidence identifies neuroinflammation as a central mechanism underlying the onset and progression of neurodegenerative diseases and CNS injury, with aging serving as a major risk factor through chronic, low-grade inflammation. Although miRNAs have been increasingly implicated in the regulation of neuroinflammatory processes during aging, neurodegeneration, and CNS injury, their diagnostic and therapeutic potential remains incompletely understood. Addressing this gap, this Research Topic brings together current advances and emerging perspectives on the diverse roles of miRNAs in neuroinflammation, aging, and neurological diseases.

Spinal cord injury (SCI) remains a grave disability with complex underlying pathophysiology. Previous studies have indicated that the NLRP3 inflammasome drives pyroptosis and plays a significant role in promoting SCI (1, 2). Alongside this, decreased levels of the anti-inflammatory and anti-apoptotic miRNA, miR-128-3p (3), have been reported in microglia in an SCI model (4). Based on these previous studies, Yang et al. investigated a pyroptosis signaling pathway modulated by miR-128-3p in SCI. They confirmed that SCI decreased expression levels of miR-128-3p in the spinal cord tissues in their SCI mouse model as well as in two different Gene Expression Omnibus SCI data sets that contain non-coding RNA expression profiles. They show that intrathecal injection of agomir-miR-128-3p suppresses oxidative stress, pyroptosis and inflammation and improves functional recovery in SCI mice. They confirmed TXNPI as a direct target of miR-128-3p by a luciferase reporter assay and *in vivo* immunohistochemistry. These observations were also confirmed in an SCI cell model (LPS-treated BV2 microglial cells) by transfection of miR-128-3p mimics. By performing the series of elegant *in vitro* and *in vivo* experiments involving miR-128-3p promoter-driven luciferase activity, ChIP qRT-PCR assays, and overexpression of FOXO3 in the SCI cell model, they clearly demonstrated that FOXO3 is a transcription factor that drives expression of miR-128 in these SCI models. They have identified the FOXO3/mir-128-3p/TXNIP/NLRP3-mediated pyroptosis pathway as a potential therapeutic target for SCI.

MiR-34a is also involved in inflammatory processes (5) and is overexpressed in the brains of individuals with Alzheimer’s disease (AD) (6). It has been implicated in cancer, metabolic diseases, and cardiovascular diseases (7, 8), all of which are associated with an altered AD risk (9–11). Additionally, miR-34a levels increase in both the blood and brain with aging (12), and it has been proposed as a promising diagnostic biomarker for AD (13). To investigate its role in AD pathophysiology, Yang et al. generated Tg-SwDI mice with constitutive miR-34a deficiency. Morris water maze testing showed that Tg-SwDI mice exhibited impaired learning compared with non-transgenic littermates, while miR-34a deficiency improved spatial long-term memory in both genotypes. Despite this cognitive improvement, miR-34a deficiency increased activated microglia and cortical soluble Aβ40, as well as insoluble Aβ40 and Aβ42, in Tg-SwDI mice (soluble Aβ42 was undetectable by ELISA). This apparent discrepancy was reconciled by a reduced Aβ42/Aβ40 ratio together with increased expression of the pre- and postsynaptic proteins (synaptophysin and PSD-95, respectively). These findings are consistent with previous studies showing that the Aβ42/Aβ40 ratio, rather than the absolute abundance of Aβ peptides, is a more important determinant of neurotoxic Aβ conformations (14). Bulk RNA-seq analysis of hippocampal tissue further revealed that miR-34a deficiency upregulated genes involved in type I interferon signaling, suggesting induction of interferon-responsive microglia (IRM). These findings indicate that miR-34a contributes to AD pathogenesis by modulating Aβ homeostasis and microglial activation, highlighting it as a potential therapeutic target.

Zanghi et al. investigated a rigorously characterized cohort of treatment-naïve patients in the earliest stages of definitive relapsing-remitting multiple sclerosis (RRMS) to identify cerebrospinal fluid (CSF) biomarkers associated with early disease pathogenesis. The study quantified cell-free CSF levels of several microRNAs previously proposed as candidate biomarkers for MS. Among these, miRNA-106a-5p was significantly upregulated in patients with RRMS compared with individuals with other neurological disorders (OND). Furthermore, CSF miRNA-106a-5p levels demonstrated a strong positive correlation with the number of oligoclonal bands (OCBs), a hallmark of intrathecal immunoglobulin synthesis and a well-established indicator of central nervous system immune activation in MS. Bioinformatic and functional analyses have implicated miRNA-106a-5p in key immune regulatory pathways, including transforming growth factor-β (TGF-β), nuclear factor kappa B (NF-κB), and phosphoinositide 3-kinase/protein kinase B (PI3K-AKT) signaling. Nevertheless, the precise mechanistic contribution of miRNA-106a-5p to MS pathogenesis remains to be fully elucidated. These findings identify miRNA-106a-5p as a promising early CSF biomarker for RRMS.

In Mini Review, Zabalza et al. present a concise review on recent progresses in our understanding of the roles of miRNAs in MS and provided their future perspective on miRNA therapeutics. The review begins with succinct description of miRNA biogenesis, nomenclature, biological function, and two different forms of miRNAs (cell-free and exosomal miRNAs) in body fluids. This general description of miRNAs is followed by summary of our current knowledge on MS pathophysiology, clinical classification, diagnosis and therapy. These general descriptions facilitate readers’ understanding of roles of miRNAs in MS pathogenesis, biomarker, and potential therapy. For cell-free miRNAs, the review found the pathogenic roles of about a dozen miRNAs in MS and identified biomarker potentials for diagnosis, prognosis and drug response in more than a dozen miRNAs. For exosomal miRNAs, the pathogenetic roles of 4 different miRNAs and a panel of 9 miRNAs discriminating relapsing remitting MS from progressive forms of MS (15) are reviewed. Additionally, miR-367-3p-containing exosomes derived from bone marrow mesenchymal stem cells are described as a potential therapeutic agent since animal models of MS are successfully treated with the agent (16). For future directions, Zabalza et al. describe developing technical approaches, limitations, and challenges of miRNA therapeutics that aim at restoring altered expression levels of miRNAs in diseases. Such approaches are generally applicable to various diseases including MS.

In summary, this Research Topic comprises three original research articles and one review, which delve into protective and pathogenic roles of miRNAs, their involvement in neuroinflammation, apoptosis and biomarker discovery, as well as their therapeutic potential. Together, these contributions highlight the broader promise of miRNAs as both biomarkers and therapeutic targets for neurological diseases and aging.
